# RNF14 is a regulator of mitochondrial and immune function in muscle

**DOI:** 10.1186/1752-0509-8-10

**Published:** 2014-01-29

**Authors:** Aaron B Ingham, Simone A Osborne, Moira Menzies, Suzie Briscoe, Wei Chen, Kritaya Kongsuwan, Antonio Reverter, Angela Jeanes, Brian P Dalrymple, Gene Wijffels, Robert Seymour, Nicholas J Hudson

**Affiliations:** 1 CSIRO Animal, Food and Health Sciences, 306 Carmody Road, St. Lucia, Queensland, Australia; 2 School of Biomedical Sciences, University of Queensland, St. Lucia, Queensland, Australia

**Keywords:** Mitochondria, Inflammation, RNF14, Muscle, Networks

## Abstract

**Background:**

Muscle development and remodelling, mitochondrial physiology and inflammation are thought to be inter-related and to have implications for metabolism in both health and disease. However, our understanding of their molecular control is incomplete.

**Results:**

In this study we have confirmed that the ring finger 14 protein (RNF14), a poorly understood transcriptional regulator, influences the expression of both mitochondrial and immune-related genes. The prediction was based on a combination of network connectivity and differential connectivity in cattle (a non-model organism) and mice data sets, with a focus on skeletal muscle. They assigned similar probability to mammalian *RNF14* playing a regulatory role in mitochondrial and immune gene expression. To try and resolve this apparent ambiguity we performed a genome-wide microarray expression analysis on mouse C2C12 myoblasts transiently transfected with two *Rnf14* transcript variants that encode 2 naturally occurring but different RNF14 protein isoforms. The effect of both constructs was significantly different to the control samples (untransfected cells and cells transfected with an empty vector). Cluster analyses revealed that transfection with the two *Rnf14* constructs yielded discrete expression signatures from each other, but in both cases a substantial set of genes annotated as encoding proteins related to immune function were perturbed. These included cytokines and interferon regulatory factors. Additionally, transfection of the longer transcript variant 1 coordinately increased the expression of 12 (of the total 13) mitochondrial proteins encoded by the mitochondrial genome, 3 of which were significant in isolated pair-wise comparisons (*Mt-coxII*, *Mt-nd2* and *mt-nd4l*). This apparent additional mitochondrial function may be attributable to the RWD protein domain that is present only in the longer RNF14 isoform.

**Conclusions:**

RNF14 influences the expression of both mitochondrial and immune related genes in a skeletal muscle context, and has likely implications for the inter-relationship between bioenergetic status and inflammation.

## Background

We are interested in understanding the regulation of muscle metabolism and its inter-relationships with development, exercise and ageing. A particular focus is the regulation of mitochondrial content which is reported to impact on metabolic syndrome in humans [1] and rats [2], feed efficiency in livestock [3] and mammalian ageing [4]. Mitochondrial content also contributes to athletic performance [5] and post-mortem meat quality [6] through the connection to muscle fibre type. The physiological connection between inflammation and muscle biology in the context of training, muscle remodelling and ageing is also of interest. For example, during exercise, muscle is routinely subject to various stressors, such as mechanical damage, hypoxia and pH decline that set in motion a pro-inflammatory cascade [7-9] which has implications for tissue remodelling. Previously, using a network science approach in cattle [10] we identified the product of the ring finger protein (*RNF14*) gene (aliases *ARA54*, *TRIAD2*), an incompletely characterised transcriptional regulator, as a factor that might influence not only mitochondrial transcription and function but also immune function. In this study we aimed to explore and validate the accuracy of this reverse-engineering under the tightly controlled experimental conditions afforded by *in vitro* cell culture.

A major foundation of our predicted function of the RNF14 protein was a bovine co-expression network [10]. Various metabolic and developmental processes were prioritised for further scrutiny on the basis of forming cohesive co-expression network gene sets or ‘modules.’ To build the network, bovine muscle sampled at different times during pre- and post-natal development, between genetically divergent breeds and following nutritional intervention, were subject to microarray analysis. By hunting in the module of interest for transcriptional regulators (DNA binding transcription factors and co-factors), or asking the related question “which transcriptional regulator has the highest absolute, average correlation to all the genes in the module?”, we generated a ranked list of regulators predicted to control the processes in question.

These approaches correctly identified groups of genes already known to play a role in mammalian skeletal muscle biology, including master regulators of the cell cycle (*E2F1*), fast twitch muscle development (*SIX1*) and mitochondrial biogenesis (*ESRRA*) [10]. Several regulatory molecules of unassigned or poorly documented function were also implicated in some of these processes, and they became candidates for future gene function validation efforts. While most genes are clearly defined within the network, one prominent gene, Ring Zinc Finger 14 (*RNF14*) a transcriptional co-activator known to bind the androgen receptor [11], gave apparently ambiguous results. Namely, it was assigned likely roles in two different processes by our analysis – immune function and mitochondrial function [10]. Given that the majority of connections in the co-expression network are positive, the prediction is that an increase in the activity of the ‘regulator’ will increase the expression of the ‘target’ genes.

A separate analysis on a different data set used a differential network strategy called Regulatory Impact Factor analysis (RIF) [12] to contrast mitochondrial-rich brown fat versus mitochondrial-poor white fat in mice. This analysis independently assigned high likelihood to a causal role for *Rnf14* in driving the phenotype differences between these cell types, also suggestive of a role in mitochondrial function and content [13]. Little is known of the function of the RNF14 protein, other than it is broadly expressed across tissues [14] and is a transcriptional co-activator that interacts with the androgen receptor transcription factor in pathways relating to sex steroid signalling. From a structural perspective, there are six *RNF14* transcript variants in humans and three in mouse, in both cases producing two different protein isoforms.

The objective of this study was to characterise the regulatory role of two RNF14 isoforms in mouse muscle. We achieved this via experimental upregulation followed by functional analysis of the subsequent genome-wide transcriptional readout. We performed a transient transfection of transcript variants encoding the two different isoforms in mouse C2C12 cells. The resultant gene expression perturbations in a number of chemokines, Interferon Regulatory Factors and related interferon signalling molecules support a role for *Rnf14* in skeletal muscle-mediated immune and inflammatory function. Additionally, the longer transcript variant 1, which encodes a protein isoform containing an RWD domain, yielded a coordinate upregulation trend of all mitochondrially-encoded mitochondrial proteins present on the array platform (12 of the total 13) reinforcing the proposed link to the mitochondrion.

## Results

### Expression constructs

**Figure 1:**
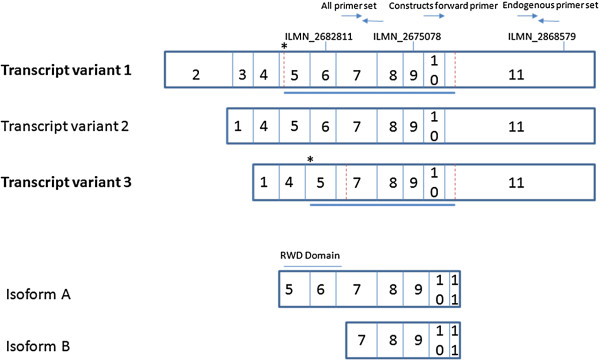
**The structures of the three *Mus musculus Rnf14 *transcript variants and their encoded proteins.** Numbers denote exons. The binding sites for the three Agilent probes, ILMN_2675078, ILMN_2682811 and ILMN_2868579 are indicated. Also highlighted are the qRT-PCR primers designed to amplify all *Rnf14* species including the transfection construct derived transcripts, and the primer set for amplifying endogenous *Rnf14* only. The forward primers for amplifying the transfection construct derived transcripts are indicated; the location of reverse primer is not shown as it was designed in vector sequence downstream of the stop codon in exon 11. The dotted red lines denote beginning and the end of the Open Reading Frames. The * denotes the position of the first base of the amplified sequence inserted into the two transfection expression constructs. Solid blue lines indicate location and size of sequence inserted into the expression constructs. The RWD domain present on isoform A (translated from the variant 1 transcript) but not isoform B (translated from the variant 3 transcript) is also highlighted. The *Rnf14* variant 2 transcript does not give rise to a protein.

PCR resulted in two differently sized *Rnf14* amplicons from mouse muscle cDNA. These amplicons were individually cloned and sequenced. In both cases the sequences exhibited >99% sequence identity to the GenBank *Rnf14* sequence. The longer sequence was 1457 bp and BLASTN aligned this sequence to the *Mus musculus Rnf14* transcript variant 1. The shorter sequence we amplified was 1306 bp, and this was aligned by BLASTN to the *Mus musculus Rnf14* transcript variant 3 (summarised in Figure 1).

*Rnf14* transcript variant 1 contains an ORF capable of producing E3 ubiquitin protein ligase RNF14 protein isoform A and *Rnf14* transcript variant 3 the ORF for E3 ubiquitin protein ligase RNF14 protein isoform B. The two isoforms are identical at the C-terminus end of the protein, while the longer isoforms has an RWD domain at the N-terminus end not present on the shorter isoform. Variant 2 does not encode a protein.

### Microarray expression measurements

The array platform used to interrogate the C2C12 response to transfection measures genome-wide transcriptional changes using 18,129 probes. This platform contains three probes predicted to bind *Rnf14*; ILMN_2675078, ILMN_2682811, ILMN_2868579. Their proposed binding sites are illustrated on Figure 1. Probe ILMN_2675078 was elevated ~3.4-fold in the variant 3 transfected cells and 1.1-fold in the variant 1 transfected cells. This 3.4-fold change made *Rnf14* the 9th most differentially expressed (DE) gene found in cells transfected with variant 3 out of the 18,129 probes with detectable signals in at least one treatment. Correcting for an overall transfection efficiency of ~10% implies that an individual transfected cell showed an increase in expression of 34-fold and 11-fold respectively.

The other two *Rnf14* probes did not report a change in expression of *Rnf14* following transfection, including ILMN_2682811, predicted to bind variants 1 and 2 but not transcript variant 3 (Table 1). It is not clear whether these 2 probes are reporting correctly. To unravel these observations and to further document the technical implentation of the *Rnf14* transfections we performed qRT-PCR on the RNA prepared from the transfected C2C12 cultures using primers designed to detect the transfection construct produced mRNA and endogenous forms of *Rnf14* mRNA.

**Table 1:** **Altered expression of *Rnf14 *** **transcripts in the *Rnf14 *** **variant 1 and variant 3 transfected C2C12 cells as reported by the three Illumina *Rnf14 *** **probes**

**Probe ID**	**Variant 3 transfected (log 2)**	**Variant 1 transfected (log 2)**	**Control cells (log 2)**	**Variant 3 transfection fold-change in *Rnf14 *** **expression**	**Variant 1 transfection fold-change in *Rnf14 *** **expression**
ILMN_2675078	10.54 ± 0.11	9.02 ± 0.10	8.78 ± 0.13	3.36	1.17
ILMN_2682811	7.44 ± 0.13	7.65 ± 0.13	7.53 ± 0.14	No change	No change
ILMN_2868579	12.73 ± 0.17	12.72 ± 0.17	12.73 ± 0.08	No change	No change

Assuming a transfection efficiency of ~10%, the fold-changes for the transfected cells are ~33-fold and 12-fold on a per cell basis (based on ILMN_2675078). The location of the probes in the different *Rnf14* transcripts is given in Figure 1.

### qRT-PCR expression measurements

To compare and contrast the *Rnf14* transcript variants in the different treatment groups, we designed a set of discriminatory qRT-PCR primers (Table 2). The ANOVA model for the analysis of Ct values from the qPCR experiments accounted for 93.03% of the total variation. The two main effects (primer and treatment) and their interaction were all highly significant (P < 0.0001).

**Table 2:** **qRT-PCR primers used to detect endogenous and transfection constructed transcribed *Rnf14 *** **mRNA species**

**Transcripts**	**5'primer**	**3'primer**
*Rnf14* all endogenous and all transfection construct	ctcaactgtccagagccaca	catggtaccaccaggctctt
*Rnf14* all endogenous only	gccccattgtgttctcaact	gagccatgatgctcttcaca
*Rnf14* variant 1 transfection construct	aattgaggaggacgacgatg	aggaactgcttccttcacga
*Rnf14* variant 3 transfection construct	aattgaggaggacgacgatg	cgtagaatcgagaccgagga
*Actb*	gtgggccgccctaggcaccag	ctctttgatgtcacgcacgatttc

Transciption from the *Actb* gene was used as a control. A schematic of the known mouse *Rnf14* mRNA species is presented in Figure 1.

The normalised expression results are summarised in Table 3. All primer sets yielded unique dissociation curves indicating the presence of *Rnf14* transcripts in the anticipated samples. The no template controls yielded no product in all cases. The transfection of *Rnf14* variant 3 was clearly evident in the variant 3 transfected cells but not the other C2C12 cultures (P < 0.0001). The transcriptional output of this expression construct was also clearly detectable in the primer set that amplifies all *Rnf14* mRNA species (i.e. endogenous and construct-based) yielding a 30-fold increase (P < 0.0001). The disparity between this result and the array fold-change is presumably attributable to the enhanced sensitivity of qRT-PCR.

**Table 3:** **Expression (Ct LSM) of each *Rnf14 *** **mRNA species and beta actin based on qRT-PCR of variant 1 and variant 3 transfected C2C12 cells**

**Treatments**	**All 3 endogenous and both constructs**	**All 3 endogenous**	**Variant 3 construct**	**Variant 1 construct**	**B actin**
Control	24.99	26.59	31.93 (no amplification)	35.6 (no amplification)	23.16
Transfected variant 3	20.17	26.73	19.41	34.19 (no amplification)	23.36
Transfected variant 1	24.89	26.92	30.32 (no amplification)	29.19	23.10

The standard error is 0.447 in all cases because we performed a system-wide normalization. ‘No amplification’ denotes multiple dissociation peaks i.e. non-specific amplification.

Similarly, the transfection of the *Rnf14* variant 1 was also clearly detectable in the variant 1 transfected cells but not the other cell cultures (P < 0.0001). The expression by the construct was not as effective as for variant 3 and was not reflected in increased amplification by the all *Rnf14* species (endogenous plus construct based) primer set, presumably because it forms a much more modest proportion of all the *Rnf14* mRNA species in the system. There was very little variability in native *Rnf14* expression (i.e. all three endogenous variants summed) across the three treatment groups. Overall, these findings imply the global transcriptional readout in the transfected C2C12 cells described by the microarray platform can be attributed to the impact of the transfection of the *Rnf14* variants.

### Functional enrichment analyses

With regard to the microarray analysis, a mixed-model normalisation procedure was applied to the raw intensity values, as previously described [15]. Clustering on columns discriminates the treatments based on global considerations of the gene expression patterns. The visualisation of the clustering analysis indicated that two of the six control samples were outliers and hence were removed for the subsequent DE analysis. Overall, the global gene expression patterns in the two control groups (no transfection and empty construct transfection) could not be discriminated from each other. Consequently, the gene expression values for the two control groups were combined to form a single control for the purposes of computing DE between controls and treatments. The *Rnf14* variant 3 and 1 transfected cells were both clustered separately to the controls (more so) and separate to each other (less so).

We computed a list of DE genes (Tables 4 and 5) as previously described [16], performing the analysis at the probe level. To explore the genome-wide expression output for functional enrichment we determined statistically significant DE for each treatment contrast. We then submitted the DE lists versus a background list of all genes present on the array to the *GOrilla* webtool [17], which uses hypergeometric statistics to determine functional enrichments.

**Table 4:** **The top 10 most upregulated genes following transfection and expression of *Rnf14 *** **variant 3 and variant 1 transcripts in C2C12 cells**

***Rnf14 *** **variant 3 versus controls**	**Gene ontology**	**Fold change (Log2)**
*Ifit3*	Interferon induced protein	2.22
*G1p2*	Interferon induced protein	2.20
*2510004L01Rik (RSAD2)*	Interferon inducible	2.09
*Oasl2*	2'-5'-oligoadenylate synthetase activity	1.97
*2310061N23Rik (Ifi27l2a)*	Interferon inducible	1.96
*Ccl5*	Chemokine ligand 5	1.91
*AI481100 (Irgm2)*	Interferon induced GTPase	1.84
*Usp18*	A member of the deubiquitinating protease family of enzymes	1.76
*Rnf14*	This protein interacts with androgen receptor (AR) and may function as a coactivator that induces AR target gene expression	1.75
*Gbp4*	Induced by interferon and hydrolyzes GTP	1.73
***Rnf14 *** **variant 1 versus controls**	**Gene ontology**	
*Plac8*	Placenta-specific, little known	2.07
*G1p2*	Interferon induced protein	1.99
*Oasl2*	2'-5'-oligoadenylate synthetase activity	1.82
*Ccl5*	Chemokine ligand 5	1.68
*LOC223672 (Apol9a)*	Apolipoprotein, little known	1.65
*2510004L01Rik*	Interferon inducible	1.61
*2310016F22Rik (Apol9b)*	Apolipoprotein, little known	1.54
*ifit3*	Interferon induced protein	1.42
*Gbp4*	Guanylate-binding proteins induced by interferon	1.30
*Oasl1*	2'-5'-oligoadenylate synthetase activity	1.21

Genes may be represented by more than one probe. The controls are a combination of untransfected cells and cells transfected with an empty construct.

**Table 5:** **The top 10 most downregulated genes following transfection and expression of *Rnf14 *** **variant 3 and variant 1 transcripts in C2C12 cells**

***Rnf14 *** **variant 3 versus controls**	**Gene ontology**	**Fold change (Log2)**
*Dcn*	The encoded protein is a small cellular/pericellular matrix proteoglycan	−1.96
*Myl1*	This gene encodes a fast myosin alkali light chain	−1.76
*Spon2*	Extracellular matrix protein	−1.63
*Prelp*	The protein encoded by this gene is a leucine-rich repeat protein present in connective tissue extracellular matrix	−1.57
*Dcn*	The encoded protein is a small cellular or pericellular matrix proteoglycan	−1.50
*Mylk*	This gene, a muscle member of the immunoglobulin gene superfamily, encodes myosin light chain kinase which is a calcium/calmodulin dependent enzyme	−1.24
*Tgm2*	Transglutaminases are enzymes that catalyze the crosslinking of proteins by epsilon-gamma glutamyl lysine isopeptide bonds	−1.20
*Tnnt1*	This protein is the slow skeletal troponin T subunit	−1.18
*Htra3*	Proteolysis	−1.17
*Mylpf*	Fast skeletal muscle	−1.16
***Rnf14 variant 1 versus controls***	**Gene ontology**	
*Dcn*	The encoded protein is a small cellular or pericellular matrix proteoglycan	−0.88
*Dcn*	The encoded protein is a small cellular or pericellular matrix proteoglycan	−0.75
*Myl1*	This gene encodes a fast myosin alkali light chain	−0.67
*Prelp*	The protein encoded by this gene is a leucine-rich repeat protein present in connective tissue extracellular matrix	−0.67
*Spon2*	Extracellular matrix, innate immune response	−0.62
*Col6a2*	Collagens are extracellular matrix proteins and have a triple-helical domain	−0.60
*Enpp1*	The encoded protein is a type II transmembrane glycoprotein	−0.57
*1190002H23Rik (Rgc32)*	Cell cycle	−0.55
*Islr*	Immunoglobulin superfamily containing leucine-rich repeat	−0.54
*C1qtnf3 (Cors)*	Beta oxidation, peroxisomal metabolism	−0.53

Genes may be represented by more than one probe. The controls are a combination of untransfected cells and cells transfected with an empty construct.

With regard to upregulation following over-expression of the shorter variant 3, “response to biotic stimulus”, “chemokine activity” and “extracellular region” gave *P*-values of 8.44E^-12^, 2.93E^-7^ and 7.4E^-14^ for the Process, Function and Component ontological levels respectively.

With regard to upregulation following over-expression of the longer variant 1, the top functional enrichments were “immune response,” chemokine activity” and “extracellular region” with hypergeometric P-values of 1.98 E^-9^, 3.83 E^-6^ and 2.06 E^-10^.

Various extracellular region components were clearly among the most downregulated genes following transfection with both variants (Table 5). The enrichment was highly significant in both cases, but more significant in the transcript 3 (*P* = 3.69 E^-19^) compared to the transcript 1 transfected cells (*P* = 1.32 E^-14^).

**Figure 2:**
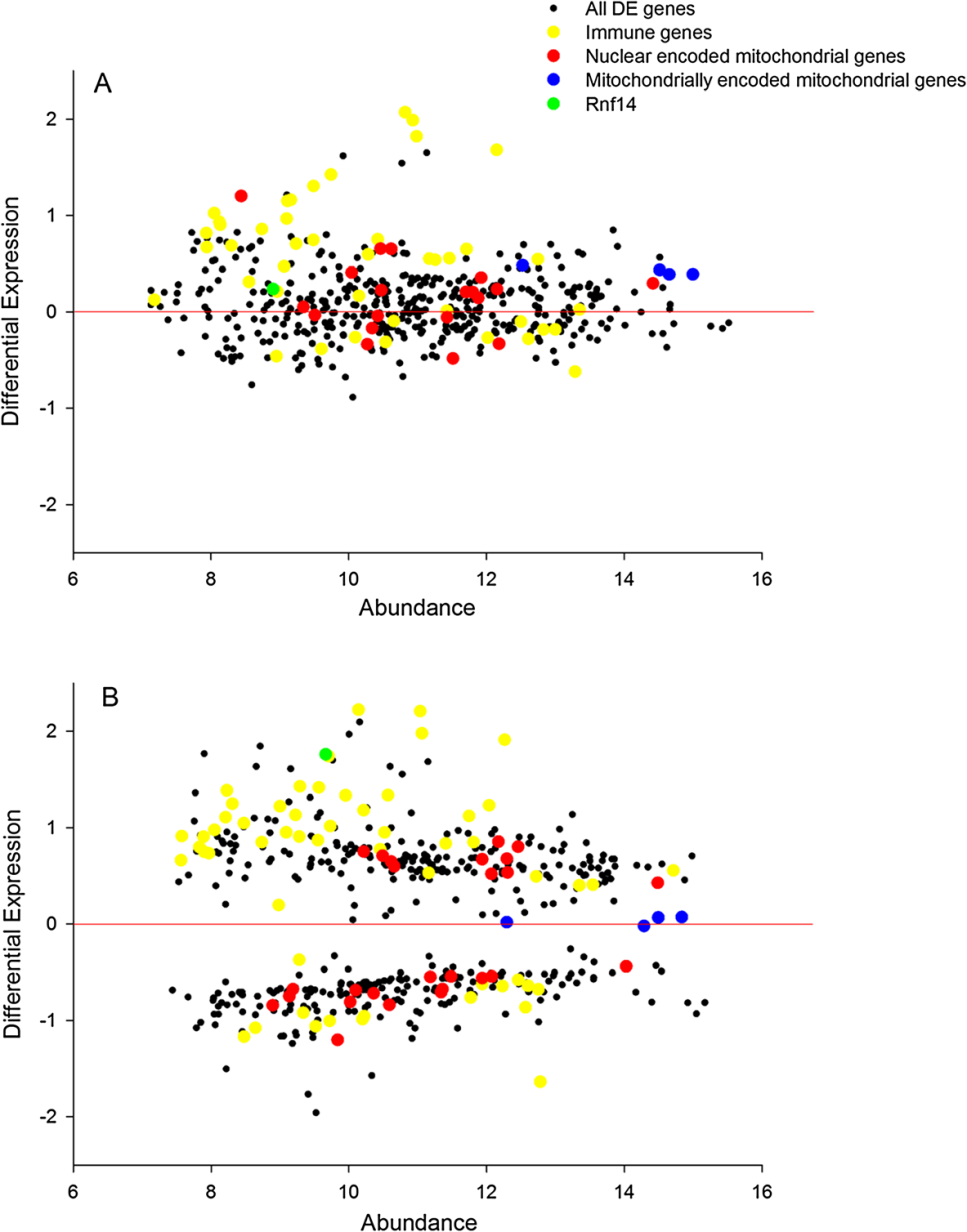
**The effect of transfection of the *Rnf14 *variant 1 and 3 transcripts on genome-wide gene expression in C2C12 cells.** Panel **A** Transfection of the *Rnf14* transcript variant 1 on genome-wide gene expression supports a functional role in mitochondrial and immune function. The significant upregulation of several mitochondrially encoded mitochondrial proteins is noteworthy. Panel **B** Transfection of *Rnf14* transcript variant 3 on genome-wide gene expression supports a functional role in immune function. The *Rnf14* probe reporting these data is ILMN_2675078.

Contrasting effects of the variant 1 transfection with that of the variant 3 transfection yielded similar functional enrichments of “response to biotic stimulus” (3.94E^-8^), signal transducer activity (2.15E^-4^) and extracellular region (1.77E^-5^). The identity of the perturbed genes is further illustrated on Figure 2 and tabulated on Tables 4 and 5. The normalised mean expression results for the entire data set are in Additional file 1. Transfection with the empty construct could not be discriminated from untransfected cells, ruling out the possibility that these responses are an experimental artefact relating to the presence of the construct. The overall spread of the perturbed transcripts is greater (4-fold DE in both directions) in the variant 3 transfected cells, which may reflect the greater abundance of the variant 3 based construct.

**Figure 3:**
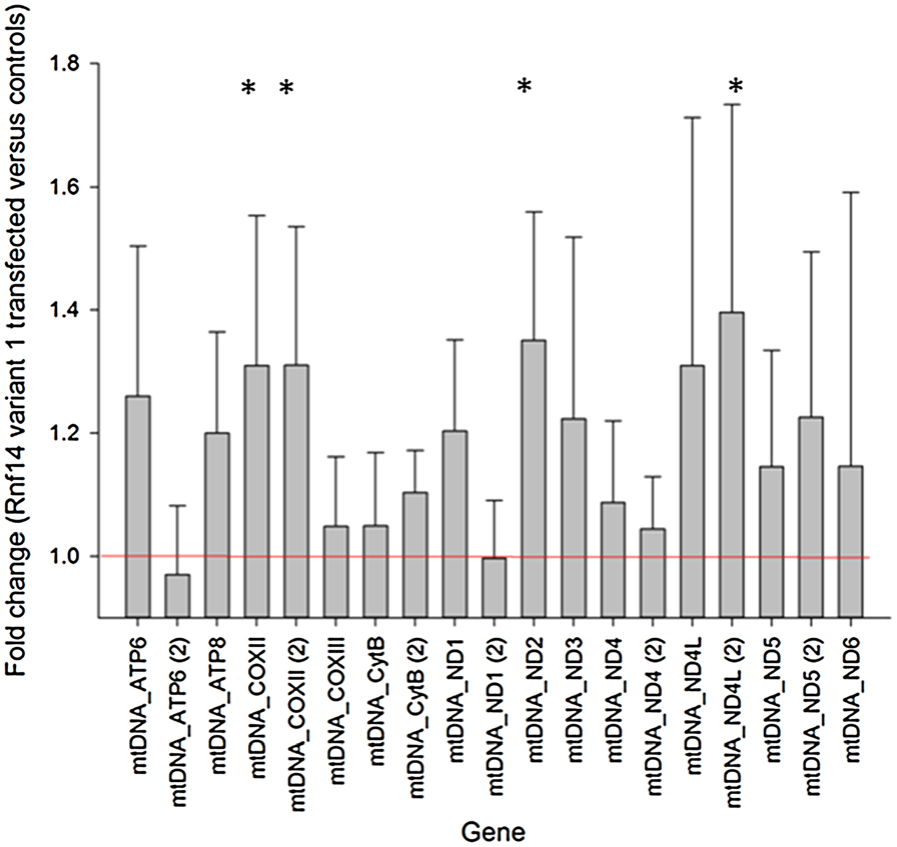
**The effect of transfection with *Rnf14 *variant 1 on gene expression of the mitochondrially-encoded mitochondrial proteins.** There are probes representing 12 of the 13 mitochondrially encoded mitochondrial genes. At least one probe representing each gene indicates a substantial trend of upregulation.

We also computed a modified DE metric called Phenotypic Impact Factors (PIF) [12,13], a product of the average abundance of the gene and its DE. We have previously found that this accounts for the increased noise of the rarer transcripts and increases the sensitivity for detecting DE of the more abundant transcripts [12]. Figure 3 illustrates those genes either DE or awarded a high PIF score in at least one of the two transfections. Immune and mitochondrial genes are highlighted based on functional annotations performed by importing the list into the DAVID web tool [18]. Immune genes and nuclear-encoded mitochondrial genes are prominent among the DE genes but the direction of change is not consistent.

On the other hand, among the significant PIF transcripts in the cells transfected with the long transcript variant are three of the 13 mitochondrially-encoded mitochondrial proteins (*Mt-coxII*, *Mt-nd2* and *Mt-nd4l*). Moreover, a deeper exploration shows that all the mitochondrially-encoded genes represented on the array (12 of the 13) display a coherent trend of upregulation in the variant 1 transfected cells based on at least one probe (Figure 3; Table 6; Additional file 2). The standard error bars for Figure 3 were calculated using the standard curve method [19]. A subset of these mitochondrially encoded genes that are significantly DE as assayed in pairwise comparisons are also highlighted on Figure 2. The upregulation of mitochondrially-encoded mitochondrial proteins was not observed in the *Rnf14* variant 3 transfected cells (Additional file 2). The full list of DE and differentially PIF genes can be found in Additional file 3.

**Table 6:** **The absolute expression levels of the mitochondrially-encoded mitochondrial proteins in the *Rnf14 *** **variant 1 transfected cells versus controls**

**Gene**	**Normalised mean expression log 2 (transfected variant 1)**	**Normalised mean expression log 2 (controls)**
Mt-coxII*	15.19 ± 0.09	14.80 ± 0.20
Mt-coxII*	14.84 ± 0.09	14.45 ± 0.21
*Mt-coxIII*	15.78 ± 0.10	15.71 ± 0.03
*Mt-atp6*	14.76 ± 0.04	14.42 ± 0.23
*Mt-atp6*	15.75 ± 0.10	15.79 ± 0.03
*Mt-atp8*	15.48 ± 0.05	15.21 ± 0.15
*Mt-cytB*	15.66 ± 0.08	15.59 ± 0.08
*Mt-cytB*	15.87 ± 0.05	15.73 ± 0.04
*Mt-nd1*	15.51 ± 0.06	15.24 ± 0.13
*Mt-nd1*	15.93 ± 0.09	15.93 ± 0.01
Mt-nd2*	14.73 ± 0.06	14.29 ± 0.19
*Mt-nd3*	8.45 ± 0.17	8.16 ± 0.23
*Mt-nd4*	15.90 ± 0.06	15.84 ± 0.05
*Mt-nd4*	15.66 ± 0.07	15.54 ± 0.10
Mt-nd4l*	12.76 ± 0.16	12.28 ± 0.29
*Mt-nd4l*	11.37 ± 0.13	10.98 ± 0.37
*Mt-nd5*	12.52 ± 0.05	12.23 ± 0.25
*Mt-nd6*	7.92 ± 0.37	7.72 ± 0.23
*Mt-nd5*	7.94 ± 0.07	7.74 ± 0.17

*Denotes pairwise significant differences at the *P* < 0.01 level.

### Motif analysis and bioinformatics

We next attempted to identify regulatory motifs that are conserved among the RNF14 responsive genes in an attempt to determine the cellular pathway linking *Rnf14* upregulation to the observed mitochondrial and inflammatory output. Hunting for conserved Transcription Factor Binding Sites 1000 bp upstream of the DE genes using Whole Genome RVista for mouse, enriched for *Hfh3* in both the variant 1 (P = 9.29-log_10_) and variant 3 transfection experiments (P = 6.57-log_10_).

We also undertook a combination of bioinformatic analyses and literature mining. A protein motif analysis of RNF14 within UNIPROTKB indicated the presence of 1) an N-terminal destruction box which could act as a recognition signal for ubiquitin proteosome degradation and 2) RING type zinc finger essential for interaction with UBE2E2.

## Discussion

In an attempt to infer the functional role of the *RNF14* protein, previously found to be co-expressed with mitochondrial and immune genes in developing bovine *longissimus* muscle, we transfected each of two *Rnf14* transcript variants into a mouse myoblast cell line. Interestingly, and despite low expression levels of that transcript, transfection with transcript variant 1 culminated in a significant upregulation in the transcription of three of the 13 mitochondrially-encoded mitochondrial genes (i.e. *Mt-nd4L*, *Mt-coxII* and *Mt-nd2*) [12]. Furthermore, while we only have microarray expression data for 12 of the 13 of these genes (*Mt-coxI* is missing) a deeper exploration shows that the remaining nine all display a modest but coherent trend of upregulation. The direction of this observation is consistent with the initial network connections being based on positive rather than negative co-expression values. While the fold-change is only 1.1 to 1.4-fold, all these transcripts are very abundantly expressed which provides a favourable signal to noise ratio for reliable detection. The array does not report on the 22 mitochondrially encoded tRNAs and two ribosomal RNAs that make up the remaining transcriptional output of the mammalian mitochondrion, which encodes 37 different genes in total.

The expression of a number of nuclear-encoded mitochondrial proteins was also upregulated following transfection of *Rnf14* variant 1 (Figure 2). For example, *Cmpk2* (alias *Tyki*) (2.4-fold up regulated) is a nucleoside monophosphate kinase that localises to the mitochondria and has previously been found to be tightly correlated with macrophage activation and inflammation [20]. A very recent publication has documented *Rnf14* as a positive regulator of canonical Wnt signalling in human cells [21], with canonical Wnt signalling previously reported to be a potent activator of mitochondrial biogenesis [22]. This recent body of work clearly complements our findings linking *Rnf14* with mitochondrial physiology.

While the broad transcriptional impact of *Rnf14* variant 1 transfection on our samples was in line with our functional prediction, there were some interesting deviations. Firstly, the nuclear and mitochondrially-encoded mitochondrial proteins occupy distinct parts of the original bovine muscle co-expression network [10]. While *Rnf14* sits in the nuclear-encoded portion of the *in vivo* bovine network, transfection with variant 1 appears to exert the most coherent transcriptional influence on the mitochondrially-encoded mitochondrial proteins. By way of contrast, *Rnf14* variant 3 transfection did not lead to a detectable change on the expression of mitochondrially-encoded mitochondrial genes, despite an overabundance of the variant 3 transcript in the variant 3 transfected cells.

Both *Rnf14* variants influenced expression of genes encoding proteins relating to immune function. Unlike the mitochondrially-encoded mitochondrial proteins upregulated observed in *Rnf14* variant 1 transfected cells, genes belonging to inflammatory processes were both up- and downregulated. Prominent among the perturbed immune genes were chemokines (e.g. *Ccl2, Ccl4, Ccl5, Ccl7, Cxcl10, Cxcl12*), interferon regulatory factors and related interferon responsive and signalling genes (e.g. *Irf1, Irf7, Irf9, Isg15, Isg20, Ifit2, Ifit3, Psmb8, Usp18, Adar, Gbp2*). In humans following eccentric exercise, the *in vivo* inflammatory response includes activation of chemokines [23]. A number of these genes also imply apoptosis, a mitochondrial phenomenon [24]. These data go some way towards resolving the question posed by our apparently ambiguous (i.e. strong co-expression to both mitochondrial and immune genes) observations from the bovine muscle co-expression network, and imply that both mitochondrial and immune predictions are supported, depending on the particular transcript variant under consideration.

Both RNF14 motifs (N-terminal destruction box and RING type zinc finger) indicate some involvement in ubiquitin mediated proteolysis which ties in with apoptosis, and UBE2E2 is known to play a specific role in adaptive immunity signalling. The motif analysis shows that most of the large transcription factor motifs (Zinc fingers and RNA binding domains) of the protein reside in the C-terminus shared by both isoforms, while the missing amino acids in the shorter isoform result in the loss of an RWD domain. We hypothesise that the RWD domain accounts for the mitochondrial response observed after transfection with *Rnf14* transcript variant 1. Recent work has emphasised deep functional connections between mitochondria and innate immunity in general [25,26], and mitochondria and antiviral processing in particular [27], which is clearly of interest given the very same dual roles outlined here for RNF14.

The downregulation of a set of extracellular region and extracellular matrix transcripts following transfection with both *Rnf14* transcript variants was unexpected. Example downregulated molecules common to both transfections included multiple collagen isoforms (*Col14a1, Col6a2, Col6a1, Col8a2, Col16a1*), other matrix structural components (*Dcn, Mglap*) and matrix remodelers (*Mmp2, Adamts2*). We ascribe these observations to one of two phenomena. On the one hand, it may reinforce the transmission of the immune signals we have observed, as it has been documented that the extracellular matrix plays a crucial role in the inflammatory process [28]. Alternatively, the signal may correspond to differences in myocyte progression through proliferation and differentiation, the transition through which is known to be accompanied by various changes in matrix-mediated adhesion [29].

Interpreting the various lines of evidence linking RNF14 protein to immune and mitochondrial functions is complicated by the cross-species sources of data. The original co-expression prediction of *RNF14’*s gene function was made mainly from bovine expression data. Disentangling the various pieces of information is challenging given cattle are a non-model organism and we have incomplete knowledge of bovine functional genomics. For example, it is not clear how many bovine *RNF14* transcript variants exist in total, which clearly complicates our (co-expression) interpretation of the Agilent probe that provided the original foundation for some of the predictions.

Nevertheless, the outcome of this validation experiment supports three of our systems biology gene discovery approaches: 1) Partial Correlation and Information Theory (PCIT) [30] 2) Module-to-Regulator analysis [10] and 3) Regulatory Impact Factors [12,13]. While there is some overlap in the exact molecules present in the co-expression modules and those perturbed in this subsequent transfection experiment (*Prsmb8* and *Irf1* being common to both in an immune context), the overlap is very patchy. This implies that predictions based on considerations of co-expression or differential co-expression are perhaps best made in terms of broad function rather than specific molecules.

## Conclusions

*Rnf14*, which encodes a transcriptional co-factor, acts via two differentially spliced transcripts to modulate innate immune responses, tissue energy homeostasis and the tissue matrix. We do not know under what stimuli either transcript is produced by cells of different lineages. We have shown that gene expression information generated in a non-model organism can be used to develop hypotheses that can be validated in more conventional systems. The validation of this approach enhances the intrinsic value of many existing datasets generated in a range of species as it provides a methodology for detailed analysis of fundamental biological phenomena, such as mitochondrial transcription and biogenesis. Future work could aim to tease out whether one or more of the RNF14 protein isoforms are mitochondrially co-localised in addition to the mRNA transcripts being mitochondrially co-expressed, and/or whether *in vivo* evidence can be detected for a role in immune function and mitochondrial transcription or biogenesis to supplement the *in vitro* data we have presented.

## Methods

### Prioritisation of RNF14 for experimental analysis

Three separate lines of evidence were used to make a prediction of *RNF14*’s possible role in immune versus mitochondrial function.

Firstly, a co-expression network on developing bovine muscle samples - inferred using the PCIT algorithm – connected *RNF14* to a dense nuclear-encoded mitochondrial module (comprising the following with correlations >0.85 *MDH1, PDHA1, NDUFS2, ES1, UQCRC1, NDUFV1, MDH2, GOT2, NDUFA9, SOD2, CYCS, NDUFV2, SDHA, BRP44, ACO2, APOO*) [10]. The position of *RNF14* in the mitochondrial module of the co-expression network made a prediction about a role in mitochondrial function.

Secondly, a related co-expression based approach called Module-to-Regulator Analysis performed on the same data set showed that - in addition to its relationship to nuclear and mitochondrially-encoded genes - *RNF14* also had very high overall co-expression patterns to the members of a separate immune module (*IKZF3, PSMB9, MYO1G, PSMB8, A_73_104252, GIMAP7, A_73_109561, A_73_111087, CD48, A_73_110856, ASPA, IRF1, PTPN7, BOLA-DRA, A_73_116074, CD3G, BOLA-DRB3 and GIMAP6*) [10] (Table 7).

**Table 7:** Top 10 transcriptional regulators showing the highest absolute, average co-expression to all the genes in the bovine muscle immune module

**Transcriptional regulator (average correlation to immune module)**	**Gene ontology**
*IRF1* (0.905)	*IRF1* encodes interferon regulatory factor 1, a member of the interferon regulatory transcription factor (IRF) family. *IRF1* also functions as a transcription activator of genes induced by interferons α, β and γ. Further*, IRF1* has been shown to play roles in regulating apoptosis
*RNF14* (0.901)	This protein interacts with androgen receptor (AR) and may function as a co-activator that induces AR target gene expression
*TEAD1* (0.872)	This gene encodes a ubiquitous transcriptional enhancer factor that is a member of the TEA/ATTS domain family
*LRRFIP1* (0.849)	Phosphorylated in response to immunologic stimuli
*EPAS1* (0.845)	Myoblast cell fate commitment
*NR1D2* (0.844)	Nuclear Hormone Receptor involved in carbohydrate and lipid metabolism
*RORC* (0.841)	Regulates *IL2* expression. IL2 is an inflammatory cytokine
*PCGF5* (0.840)	Little known
*PHF12* (0.838)	Little known
*HOXD8 (*0.838)	The homeobox genes encode a highly conserved family of transcription factors that play an important role in morphogenesis

Thirdly, application of the Regulatory Impact Factor (RIF) algorithm to an entirely independent data set (comparing high-mitochondrial content brown fat versus low-mitochondrial content white fat) awarded high likelihood of phenotype causality to the RNF14 protein [13].

### Amplification, cloning and transformation of two transcript variants of RNF14

The mouse ortholog for RNF14 (GenBank accession NM_020012.1) was identified through sequence alignment to the bovine RNF14 protein. Mouse muscle cDNA was used as template for a PCR designed to amplify the full length mRNA encoding the *Rnf14* gene. To increase recognition of the first ATG by the ribosome, the forward primer incorporated the mammalian Kozak sequence (CCATGG) prior to the start of the Open Reading Frame. PCR primers also incorporated vector sequence, restriction enzyme sites used for cloning (*NcoI* at 5’ and *XhoI* 3’) and gene specific sequence. The forward primer for PCR was 5'- GAGATATGCCA*CCATGG*AGTCGGCAGAAGACCTGGAAGCCCAG-3' and the reverse primer 5'- GTGATGGTGGTG*CTCGAG*GTCGTCGTCGTCGTCTTCATCATCGT-3'.

A conventional PCR with a 50°C annealing temperature using high fidelity *Taq polymerase*, amplified a band of 1457 base pairs. This was gel purified and cloned into the pTANDEM-1 vector (EMD4 Biosciences, Merck, USA) using a fusion homologous recombination method. The resultant expression plasmid contained a His_6_ tag at the C terminus of the RNF14 gene for protein purification and detection by antibodies.

In a separate PCR reaction a shorter *Rnf14* transcript variant was also amplified (1306 bp), using the forward primer 5'-ATGTCGGCAGAAGACCTGGAAGC-3' and the reverse primer 5'-CTAGTCGTCGTCGTCGTCTTCATCATCG-3'. This PCR product was cloned into the pcDNA3.1/V5-his TOPO TA vector (Invitrogen, USA).

The two variants are referred to as *Rnf14* transcript variant 1 and *Rnf14* transcript variant 3. Following transformation into TOP10 chemically competent *E.coli*, clones were picked and diagnosed for the presence of the insert in the correct orientation using restriction digests (*Xho*I and *Nco*I) followed by gel electrophoresis. Clones predicted to contain the correct insert were sequenced from both ends of the expression construct using the BGH, T7 and pTandem1 Tandem DOWN1 primer and aligned to the GenBank mouse RNF14 sequence.

Expression constructs containing the correct sequence were midi-prepped (Qiagen) following the manufacturer’s instructions and stored at 1 μg/μl at −20°C in preparation for transfection. The Qiagen midi-prepping protocol removes LPS and provides transfection-ready reagents.

A control construct containing no insert (hereon referred to as “empty”) was also produced and subsequently used for transfection. A separate control was derived from untransfected cells.

### Cell culture and transfection conditions

The murine myoblast cell line, C2C12, was obtained from the American Type Culture Collection and cultured in DMEM supplemented with penicillin/streptomycin and 10% fetal bovine serum. Transfection conditions for the C2C12 cells (passage numbers 15 – 20) were explored for a range of confluences and Lipofectamine 2000 (Invitrogen) transfection reagent: DNA ratios. A pEGFP.C3 vector containing Green Fluorescent Protein (GFP) was used to visualise transfection efficiency under a Zeiss AX10 fluorescence microscope fitted with an Axiocam camera. As previously reviewed [31], we found that C2C12 cells transfected with a low transfection efficiency of ~10%. Optimal cell number was found to be 5 × 10^4^ cells/well transfected with 5 μl of Lipofectamine 2000 and 800 ng DNA per well for 48 hours.

The experiment was run in two 24-well Geltrex-coated plates using the optimised procedure. Cells were cultured for 24 hours prior to transfection. At the time of transfection the cells were ~50% confluent. Two independent controls were used, one with no transfection and the other through transfection of the empty construct.

Twelve independent wells were run for each of the four treatment groups (no transfection, empty construct, *Rnf14* short transcript variant and *Rnf14* long transcript variant) giving a 48 well cell culture experiment in total. At 48 hours post transfection the cells from each well were harvested for RNA, while a GFP transfection run in parallel was fixed and stained to provide an estimate of transfection efficiency.

### RNA extraction and microarray hybridisation

Total RNA was independently extracted from each of the 48 cell culture wells using QIAshredder homogenates and QIAgen RNeasy columns (Qiagen), incorporating the on-column DNase treatment (Ambion) to remove genomic DNA contamination. RNA integrity and purity was assayed visually by gel electrophoresis and additionally by A_260/280_ spectrophotometry. All the RNA samples resolved into discrete 16S and 26S ribosomal RNA bands.

Because each of the four treatment groups comprised 12 independent RNA samples, these 12 were pooled into 3 major groups of 4 replicates with each replicate contributing 0.5 μg. This process yielded 12 by 2.0 μg pooled RNA samples as follows (no transfection 1, no transfection 2, no transfection 3, empty construct 1, empty construct 2, empty construct 3, short transcript 1, short transcript 2, short transcript 3, long transcript 1, long transcript 2, long transcript 3).

The 12 RNA pools were submitted to the Gene Expression Centre at the University of Queensland’s Institute for Molecular Bioscience for cDNA synthesis and hybridisation to the Illumina WG6 mouse microarray platform. This facility performed an additional set of quality checks based on possession of an RNA Integrity Number (RIN) >8 assayed on a Bioanalyzer.

### Statistical analysis

The analytical procedures to normalize the data and to identify differentially expressed genes were based on methods developed by our group and published elsewhere. In particular, we followed the methodology described in [15,32,33].

The raw data from the Illumina Microarray System contained expression signals from 45,281 probes across the 12 hybridizations.

### Initial normalization

We base-2 Log-transformed the expression signals to stabilize the variance, capturing only those probes with a “detectable” signal (P < 0.01) in at least one of the 12 hybridizations. This produced a “cleaned” dataset of 18,129 Illumina probes.

### Chip normalization

The “cleaned” dataset was subject to a within chip normalization in which each signal was normalized by subtracting the within-chip mean and dividing by the within-chip standard deviation. The chips were therefore standardized to have a mean (μ) of 0 and a standard deviation (σ) of 1, enabling data from different chips to be combined without the risk of being influenced by differences in mean or SD. This allows subsequent linear functions to be more reasonably derived, in our case computation of differential expression. The resulting normalized dataset was subjected to hierarchical cluster analyses using the PermutMatrix software [34].

### Identification of DE/DPIF genes

For the identification of DE/DPIF genes we focused on two contrasts: variant 1 versus controls and variant 3 versus controls. Following [35] a two-component normal mixture model was fitted to identify DE/DPIF genes. Parameters of the mixture model were estimated using the EMMIX-GENE software [36] and an estimated experiment-wise false discovery rate (FDR) of < 1% used as the threshold for determining which genes are DE/DPIF.

### qRT-PCR

We used Superscript III (Invitrogen) for cDNA synthesis for the 12 pools following the manufacturer’s instructions, in each case using 1ug of the same RNA stock hybridised to the microarray platforms. The resultant 20 ul of neat cDNA was diluted to make a 1:10 solution for qPCR.

Four primer sets were designed to discriminate between endogenously produced and transfection construct generated *Rnf14* mRNA species (Table 2). In each case, Primer3 software was used for primer design to amplify 100–200 bp of sequence based on a 60°C annealing temperature. We accepted the remainder of Primer 3’s default settings.

To assay expression all endogenous *Rnf14* transcripts we designed primers in the 3' UTR of exon 11 (Figure 1). This exon is shared by all variants, but will not amplify construct based transcripts because they possess vector sequence downstream of the stop codon. To assay all *Rnf14* transcripts (endogenous and from the transfection constructs) we designed primers in exons 7–8 that are shared by all the mRNA species. To assay transcription relating to the two expression constructs (forward primer visualised on Figure 1) we designed the forward primer prior to the stop codon in exon 11 and the reverse primer in the downstream vector sequence.

For the qRT-PCR assays we used a Sybr Green master mix with 900 nM of each primer and a constant amount of cDNA run on an Applied Biosystems ViiA7 thermal cycler (Forster City, CA, USA) in 10 μl reactions. qPCR reactions were run in triplicate with a no template control for each primer set. Expression was normalised using beta-actin as a reference gene. Because the empty construct and untransfected cells could not be discriminated by microarray, we used the empty construct as the control RNA’s for qRT PCR.

### qRT PCR statistical analysis

Following previously described approaches [37] statistical analysis was carried out using SAS software 9.2 (SAS Institute, Cary, NC), using triplicate Ct values per sample. In particular the Ct values were normalized by fitting an analysis of variance (ANOVA) model that accounted for “within primer across treatment” and “within treatment across primer” variation. A general linear model approach was used consisting of the following: Expression (Ct) ~ Primer + Treatment + Primer*Treatment + Error. Finally, for each Primer, the corresponding contrast of least-square means for Ct values in the Treatment of interest was used to ascertain differential expression.

## Competing interests

The authors declare they have no competing interests.

## Authors’ contributions

SAO, SB, WC, KK, AJ, MM and NJH performed the experiments. AR performed the statistical analyses. ABI, GW and RS helped design and interpret the experiments. BPD performed the bioinformatic analysis. NJH drafted the manuscript. RBS, GW and NJH resourced and managed the project. All authors read, contributed to, and approved the manuscript.

## Supplementary Material

Additional file 1 The normalized mean expression values (log 2) for all genes across the 12 biological replicates. Click here for file

Additional file 2 **The fold changes in the mitochondrially-encoded mitochondrial proteins following *Rnf14 *** **transfection.** Click here for file

Additional file 3 **Those genes significantly DE and DPIF following *Rnf14 *** **transfection.** Click here for file
