# Supplementary material for: RNF14 is a regulator of mitochondrial and immune function in muscle
Source: BMC Syst Biol. 2014 Jan 29;8:10. doi: 10.1186/1752-0509-8-10 (PMC3906743; doi:10.1186/1752-0509-8-10)
Supplement: Additional file 2 — The fold changes in the mitochondrially-encoded mitochondrial proteins following Rnf14 transfection. [file 1752-0509-8-10-S2.docx]

The average fold-change in expression of 12 of the 13 mitochondrially-encoded mitochondrial proteins in the two *Rnf14* transfections of C2C12 cells. Some genes are represented by more than one probe. The array does not report on *Mt-coxI*. In all genes there is a substantial trend of up-regulation in the variant 1 transfected cells for at least one probe representing that gene. This is not the case for the *Rnf14* variant 3 transfected cells. All values in excess of 1.2-fold are in **bold**.

| **Gene** | **Probe** | **DE *Rnf14* variant 1 minus control** | **DE *Rnf14* variant 3 minus control** |
| --- | --- | --- | --- |
| *Mt-coxII* | ILMN_1248039 | **1.31** | 1.05 |
| *Mt-coxII* | ILMN_2426691 | **1.31** | 1.05 |
| *Mt-coxIII* | ILMN_2454649 | 1.05 | 1.11 |
| *Mt-atp6* | ILMN_2504686 | **1.26** | 1.08 |
| *Mt-atp6* | ILMN_2470277 | 0.97 | 0.99 |
| *Mt-atp8* | ILMN_2436634 | **1.20** | 1.02 |
| *Mt-cytB* | ILMN_1239479 | 1.05 | 1.09 |
| *Mt-cytB* | ILMN_1239040 | 1.10 | 1.01 |
| *Mt-nd1* | ILMN_2469966 | **1.20** | 1.05 |
| *Mt-nd1* | ILMN_2483935 | 1.00 | 0.98 |
| *Mt-nd2* | ILMN_2434853 | **1.35** | 0.99 |
| *Mt-nd3* | ILMN_2495796 | **1.22** | 1.05 |
| *Mt-nd4* | ILMN_1241022 | 1.09 | 1.01 |
| *Mt-nd4* | ILMN_2428846 | 1.04 | 1.03 |
| *Mt-nd4l* | ILMN_2512204 | **1.31** | 0.98 |
| *Mt-nd4l* | ILMN_2419660 | **1.40** | 1.01 |
| *Mt-nd5* | ILMN_2504544 | 1.14 | 1.09 |
| *Mt-nd5* | ILMN_2507810 | **1.23** | 1.09 |
| *Mt-nd6* | ILMN_1219839 | 1.15 | 1.04 |
